# Longitudinal PET Imaging to Monitor Treatment Efficacy by Liposomal Irinotecan in Orthotopic Patient-Derived Pancreatic Tumor Models of High and Low Hypoxia

**DOI:** 10.1007/s11307-019-01374-x

**Published:** 2019-09-03

**Authors:** Manuela Ventura, Nicholas Bernards, Raquel De Souza, Inga B. Fricke, Bart S. Hendriks, Jonathan B. Fitzgerald, Helen Lee, Stephan G. Klinz, Jinzi Zheng

**Affiliations:** 1 TECHNA Institute for the Advancement of Technology for Health, University Health Network, Toronto, Ontario Canada; 2 Merrimack Pharmaceuticals, Inc., Cambridge, MA USA; 3 Ipsen Bioscience, Cambridge, MA USA; 4 Institute of Biomaterials and Biomedical Engineering, University of Toronto, Toronto, Ontario Canada

**Keywords:** ONIVYDE, Liposomal irinotecan, Hypoxia, Pancreatic cancer, [^18^F]FAZA

## Abstract

**Purpose:**

Hypoxia is linked to aggressiveness, resistance to therapy, and poor prognosis of pancreatic tumors. Liposomal irinotecan (nal-IRI, ONIVYDE®) has shown potential in reducing hypoxia in the HT29 colorectal cancer model, and here, we investigate its therapeutic activity and ability to modulate hypoxia in patient-derived orthotopic tumor models of pancreatic cancer.

**Procedures:**

Mice were randomized into nal-IRI treated and untreated controls. Magnetic resonance imaging was used for monitoring treatment efficacy, positron emission tomography (PET) imaging with F-18-labelled fluoroazomycinarabinoside ([^18^F]FAZA) for tumor hypoxia quantification, and F-18-labelled fluorothymidine ([^18^F]FLT) for tumor cell proliferation.

**Results:**

The highly hypoxic OCIP51 tumors showed significant response following nal-IRI treatment compared with the less hypoxic OCIP19 tumors. [^18^F]FAZA-PET detected significant hypoxia reduction in treated OCIP51 tumors, 8 days before significant changes in tumor volume. OCIP19 tumors also responded to therapy, although tumor volume control was not accompanied by any reduction in [^18^F]FAZA uptake. In both models, no differences were observable in [^18^F]FLT uptake in treated tumors compared with control mice.

**Conclusions:**

Hypoxia modulation may play a role in nal-IRI’s mechanism of action. Nal-IRI demonstrated greater anti-tumor activity in the more aggressive and hypoxic tumor model. Furthermore, hypoxia imaging provided early prediction of treatment response.

**Electronic supplementary material:**

The online version of this article (10.1007/s11307-019-01374-x) contains supplementary material, which is available to authorized users.

## Introduction

Pancreatic cancer is the third leading cause of cancer-related death in the USA [1], and its incidence rates are continuously increasing [2, 3]. In spite of continuous advances in diagnostic tools and treatment regimes, the prognosis of pancreatic cancer remains poor. Several studies suggested a dominant role of hypoxia in the aggressiveness and resistance to therapy of these and other solid tumors [4–7]. Clinically, it is also recognized that hypoxic tumor regions are those responsible for resistance to chemotherapy and radiation therapy [8] and that hypoxia promotes metastatic development [9, 10].

Evidence of hypoxia in pancreatic cancer was first shown by Koong et al. [11] through intraoperative pO_2_ measurements in patients. More recently, Chang et al. [12] characterized a series of early passage xenografts established from pancreatectomy samples, demonstrating a correlation among hypoxia levels, aggressive growth, and metastasis formation.

Various hypoxia-specific positron emission tomography (PET) tracers have been successfully employed, clinically and preclinically, for the detection of changes in intratumoral hypoxia over time [13–16]. Most tracers are based on nitroimidazoles, which are reduced after cellular internalization and only reversed to the original compound in the presence of intracellular oxygen, thus becoming selectively trapped within hypoxic cells [17]. [^18^F]fluoromisonidazole (FMISO) was the first clinically approved hypoxia tracer [8, 18]. More recently, [^18^F]fluoroazomycinarabinoside (FAZA) was introduced due to its more rapid clearance from non-hypoxic tissues, although studies comparing the two tracers have shown comparable performances [19, 20].

The use of [^18^F]FAZA-PET imaging as a prognostic tool, with the power to predict therapeutic outcome, has been suggested by both clinical and preclinical studies [9, 10, 21]. In spite of that, only two studies have reported its use to monitor hypoxia [22, 23], and, to our knowledge, no studies have reported its use to evaluate the therapeutic response in pancreatic tumors, in which hypoxia represents a well-established therapeutic challenge. Interestingly, some chemotherapy agents have also shown the capability to modulate hypoxia [24]. Among those is liposomal irinotecan (nal-IRI, ONIVYDE®, irinotecan liposome injection, MM-398), currently approved in North America, Europe, and select Asia-Pacific countries in combination with fluorouracil and leucovorin for the treatment of patients with advanced metastatic pancreatic cancer after disease progression following gemcitabine-based therapy [25], a tumor indication characterized by low vascular density as well as numerous and pronounced hypoxic regions [26]. Nal-IRI is a highly stable liposomal nanocarrier formulation of the prodrug irinotecan, able to achieve significantly higher and extended intratumoral exposure levels of both irinotecan and its active metabolite SN-38, compared with conventional free irinotecan [27, 28]. Sustained exposure can result in reduced levels of hypoxia-regulated markers relative to reference tumors, after prolonged treatment, or as immediately as following a single-dose administration [29–31].

In this study, we explore the value of longitudinal [^18^F]FAZA-PET imaging in two orthotopic patient-derived xenograft (PDX) models of pancreatic cancer with low (OCIP19) and high hypoxia (OCIP51), following treatment with nal-IRI. The goal of this investigation is to evaluate both the therapeutic activity of nal-IRI in orthotopic pancreatic cancer models and to assess its ability to modulate hypoxia changes in tumors that have inherently high or low baseline hypoxia levels. [^18^F]FLT-PET imaging was also employed to track changes in tumor cell proliferation following nal-IRI therapy.

## Materials and Methods

### Animal Models

Details of the animal models development are provided in the Electronic Supplementary Material (ESM).

### Chemotherapy Treatment, Toxicity, and Humane Endpoints

For the treatment efficacy study, mice were randomized into (i) nal-IRI (Merrimack Pharmaceuticals, Cambridge, MA, USA) treated at 20 mg/kg (*n* = 10 per tumor model) and (ii) untreated control (*n* = 5 per tumor model) which did not receive any injection. Please refer to Suppl. Fig. 1 (see ESM) for a schematics of the therapeutic regime. Treatment toxicity was assessed by monitoring the weight of the mice over the entire experimental period. The criteria for humane endpoints were tumor volume (if ≥ 1500 mm^3^), significant constriction of surrounding organs, and loss of over 20 % of the body weight.

**Fig. 1:**
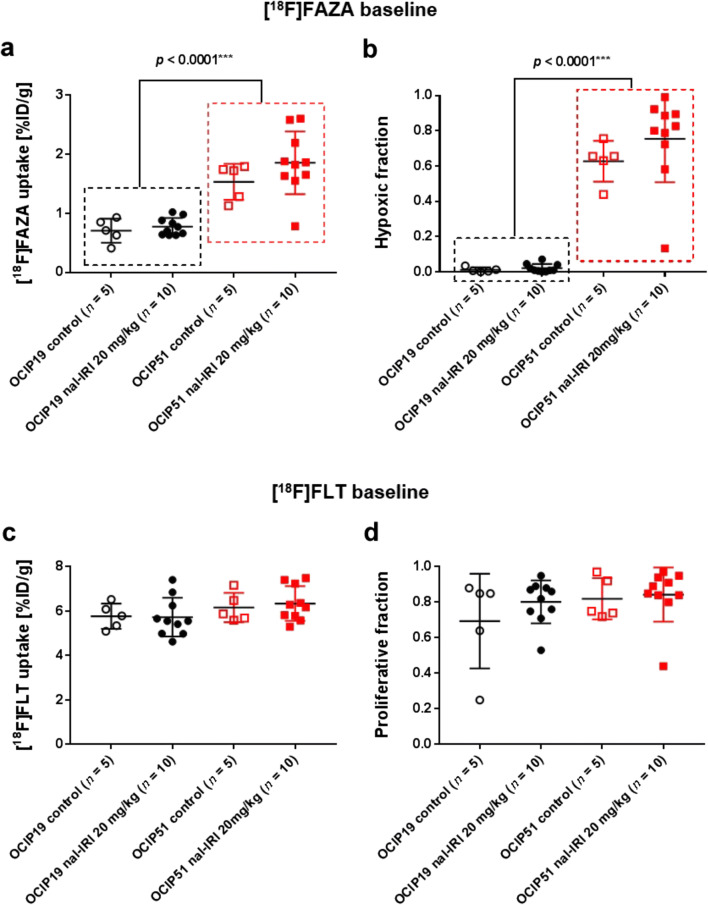
[^18^F]FAZA and [^18^F]FLT uptake characteristics of OCIP51 and OCIP19 tumors at study baseline. Pretreatment tumor [^18^F]FAZA uptake expressed as **a** mean %ID/g and as **b** hypoxic fraction. [^18^F]FLT tumor uptake, measured prior to treatment initiation, expressed as **c** mean %ID/g and **d** proliferative fraction.

### *In Vivo* Imaging

Please refer to the ESM for a detailed description of the *in vivo* imaging methods and for a schematics of the *in vivo* imaging timeline (Fig. S1).

### Image Analysis

Magnetic resonance imaging (MRI) data were analyzed using MicroView (GE Healthcare). [^18^F]FAZA and [^18^F]FLT-PET were each co-registered semi-automatically using the semi-rigid co-registration tool to corresponding CT datasets, and tumor and muscle regions of interest (ROIs) were drawn and analyzed using the Inveon Research Workplace software (IRW 4.0, Siemens). Representative CT- and MRI-based ROIs are shown in Suppl. Fig. S2 (see ESM). For the hypoxic fraction analysis (number of hypoxic tumor voxels over number of total tumor voxels), a voxel was classified to be hypoxic if its [^18^F]FAZA-PET signal was higher than an animal-specific threshold. This threshold was defined as the mean + 3 standard deviations of the [^18^F]FAZA-PET signal value measured in the muscle region of the same animal [21]. The same quantification method was used to assess the fraction of [^18^F]FLT-positive voxels within a tumor.

### Statistical Analysis

Differences between means for the different treatment groups were compared using two-way ANOVA or an independent sample non-parametric Mann-Whitney test with a confidence interval of 95 %. All statistical calculations were performed using GraphPad Prism v7.00 (GraphPad Software, La Jolla, CA, USA). Results are reported as mean ± standard deviation.

## Results

### [^18^F]FAZA and [^18^F]FLT-PET Uptake Characteristics in OCIP51 and OCIP19 Tumors at Baseline

Among a panel of 16 patient-derived pancreatic xenografts for which the degree of hypoxia has been reported, OCIP51 was among the three most hypoxic and OCIP19 among the three least hypoxic tumors. Accordingly, we chose OCIP51 to represent a high hypoxia tumor and OCIP19 as a representative low hypoxia model. The average tumor volumes on the treatment initiation day (day 0) were not found to be statistically different between the control and treatment groups. Specifically, for the OCIP19, the average tumor volumes at the time of randomization for the nal-IRI treatment and control group were 302.3800 ± 123.4298 mm^3^ and 340.0200 ± 69.5059 mm^3^ (*p* = 0.4396, n.s.), respectively. For the OCIP51, the average tumor volume at randomization for the nal-IRI treatment group was 259.2260 ± 183.3490 mm^3^ and for the control animals was 316.3960 ± 153.7385 mm^3^ (*p* = 0.5341, n.s.).

At study baseline (day 0), as shown in Fig. 1a, the OCIP51 model exhibited significantly (*p* < 0.0001) higher tumor [^18^F]FAZA uptake (1.75 ± 0.12 %ID/g, *n* = 15) compared with the OCIP19 model (0.75 ± 0.04 %ID/g, *n* = 15). Even more evident was the difference in the tumor hypoxic fraction [22, 32] (Fig. 1b), 0.02 ± 0.01 for the OCIP19 tumors *versus* 0.71 ± 0.06 for the OCIP51 tumors (*p* < 0.0001). [^18^F]FLT, a surrogate marker for cellular proliferation [33], was employed as a non-hypoxia-dependent measurement for evaluation of treatment response. Prior to treatment initiation, baseline [^18^F]FLT-PET (Fig. 1c, d) showed similar proliferation profiles for the two tumor models both in terms of mean [^18^F]FLT uptake (6.28 ± 0.70 %ID/g for OCIP51 and 5.74 ± 0.74 %ID/g for OCIP19, *p* = 0.0570) and the calculated tumor proliferative fraction (0.84 ± 0.13 for OCIP51 and 0.77 ± 0.17 for OCIP19, *p* = 0.2453). For both [^18^F]FAZA and [^18^F]FLT uptake, no statistically significant differences were found between the two groups at study baseline. Both [^18^F]FAZA and [^18^F]FLT uptake in the muscle are also shown for each group in Suppl. Fig. S3 (see ESM).

### Longitudinal Monitoring of Treatment and Efficacy

**Fig. 2:**
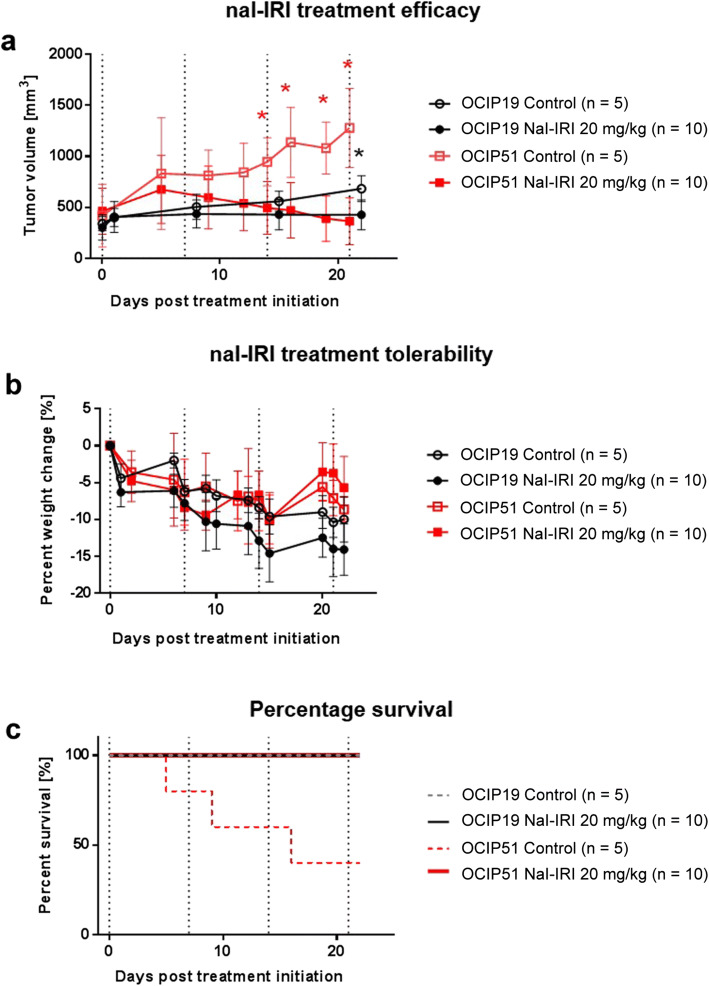
Longitudinal monitoring of nal-IRI treatment efficacy and toxicity. **a** Tumor volumes as measured by MRI. **b** Longitudinal monitoring of the animals’ body weight, showed as percentage of change relative to treatment day 0. **c** Percent survival for the four groups. Dotted vertical lines indicate time points of nal-IRI treatment administration.

As illustrated in Fig. 2a, OCIP51 and OCIP19 tumors showed statistically significant responses compared with their respective control groups as treatment with nal-IRI progressed. Specifically, treatment response was first observed on day 14 post-treatment initiation in the OCIP51 model (*p* = 0.0280) and on day 22 in the OCIP19 model (*p* = 0.0080). The delay in showing statistically significant differences between the control and treated OCIP19 tumors is in part due to the slower disease progression of the untreated tumors for this low hypoxia model (tumor volume doubling time = 27.08 days), compared with the OCIP51 model (tumor volume doubling time = 11.28 days). Both tumor models demonstrated response to nal-IRI treatment on day 21/22. For the more hypoxic OCIP51 model, the average volume at day 21 of the treated tumors (268.6 ± 133.8 mm^3^) was 4-fold smaller compared with that of the untreated controls (1085.4 ± 241.7 mm^3^), shrinking to about 72.4 ± 27.2 % of their volume at the start of the study and to 49.7 ± 20.2 % of their maximal tumor volumes reached around day 5 after treatment initiation (see Suppl. Fig. S4 in ESM for relative changes of tumor volume in the OCIP51 model). For the less hypoxic OCIP19 model, the average volume of the treated tumors (494.2 ± 149.2 mm^3^) was 69 % of that of the untreated controls (712.6 ± 127.9 mm^3^) although still 1.5-fold larger than their volume at treatment initiation.

### Treatment Tolerability and Overall Survival

All animals in this study, whether treated or untreated, lost weight over time. However, the change in body weight (Fig. 2b) relative to the day of treatment initiation was not significantly different between the treated and untreated groups for either tumor model. All OCIP19-bearing animals survived until the end of the study, whereas for the OCIP51 model, only 40 % of the untreated control mice survived to study endpoint with three out of five control mice having to be sacrificed due to excessive tumor burden (> 1500 mm^3^) on days 5, 9, and 16 post-treatment initiation, respectively (Fig. 2c).

### Nal-IRI Mediated Hypoxia Modulation in Tumor Models of High and Low Baseline Hypoxia

**Fig. 3:**
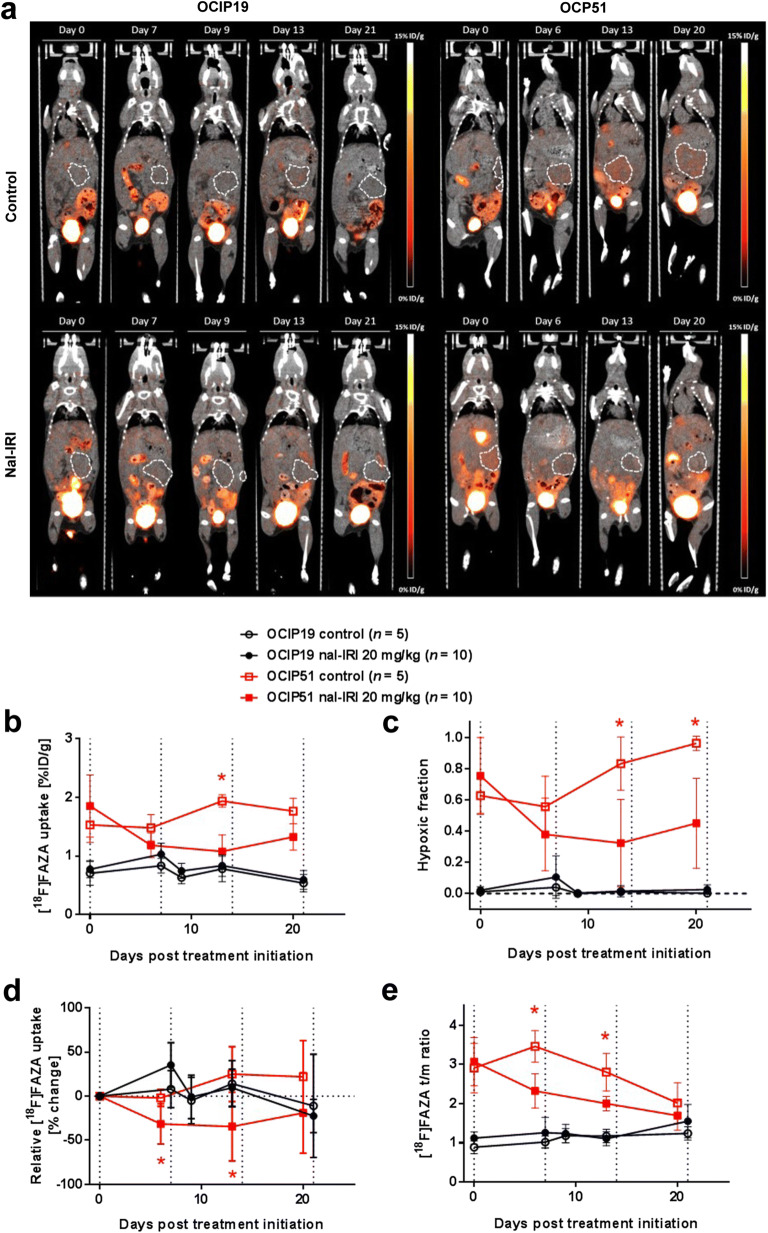
Longitudinal [^18^F]FAZA-PET imaging. **a** Representative 2D coronal PET/CT images from a control and a treated mouse for each tumor model. The tumor contours are highlighted by the white dashed lines. **b** [^18^F]FAZA uptake expressed as mean %ID/g over the entire tumor volume and as **c** tumor hypoxic fraction calculated from the PET data set. **d** [^18^F]FAZA uptake changes relative to treatment initiation day 0. **e** [^18^F]FAZA uptake expressed as tumor-to-muscle (t/m) ratio of the mean %ID/g. Asterisks denote a statistically significant difference (*p* < 0.05) between the nal-IRI-treated group and the control group. Dotted vertical lines indicate time points of nal-IRI treatment administration.

In the OCIP51 model, significant differences in tumor hypoxia between treated and control animals were observed starting 13 days post-treatment initiation (Fig. 3a), in terms of %ID/g of [^18^F]FAZA-PET uptake (Fig. 3b; *p* = 0.0070) and hypoxic fraction (Fig. 3c; *p* = 0.0280). For both measurements, a decrease in signal (36.1 % and 49.8 %, respectively) was found in treated animals at day 6, compared with their baseline values (Fig. 3b, c). These early decreases in OCIP51 tumor hypoxia were significant when plotted as relative changes in [^18^F]FAZA-PET uptake with respect to study baseline (Fig. 3d) and tumor-to-muscle signal ratios (Fig. 3e) with *p* = 0.0120 and *p* = 0.0013, respectively. At the study endpoint, [^18^F]FAZA-PET uptake in nal-IRI-treated OCIP51 animals remained significantly lower than at baseline, in terms of %ID/g (*p* = 0.0027), hypoxic fraction (*p* = 0.0147), and tumor-to-muscle ratio (*p* < 0.0001), whereas for the control group, no significant differences were found for these parameters between the two time points. In the OCIP19 model, no statistically significant difference in tumor hypoxia level was observed for any of the [^18^F]FAZA-PET-based hypoxia quantification parameters over the 3 weeks of nal-IRI treatment (Fig. 3a–e), although a positive therapeutic response was observed in terms of tumor volume differences between the treated and the untreated control groups starting on day 22. No predictive value was found when looking at individual animals in the OCIP19 model, whereas in the OCIP51, a correlation was found (Spearman correlation *ρ* = 0.6750, *p* = 0.0370) between the hypoxic fraction on day 0 and the % of tumor volume change at the study endpoint. On average, the hypoxia levels in %ID/g of [^18^F]FAZA uptake measured in the OCIP51 model were 1.89 ± 0.003 times higher compared with those in the OCIP19 tumors throughout the entire course of the study.

### Longitudinal [^18^F]FLT-PET Imaging of Nal-IRI Treatment Response

**Fig. 4:**
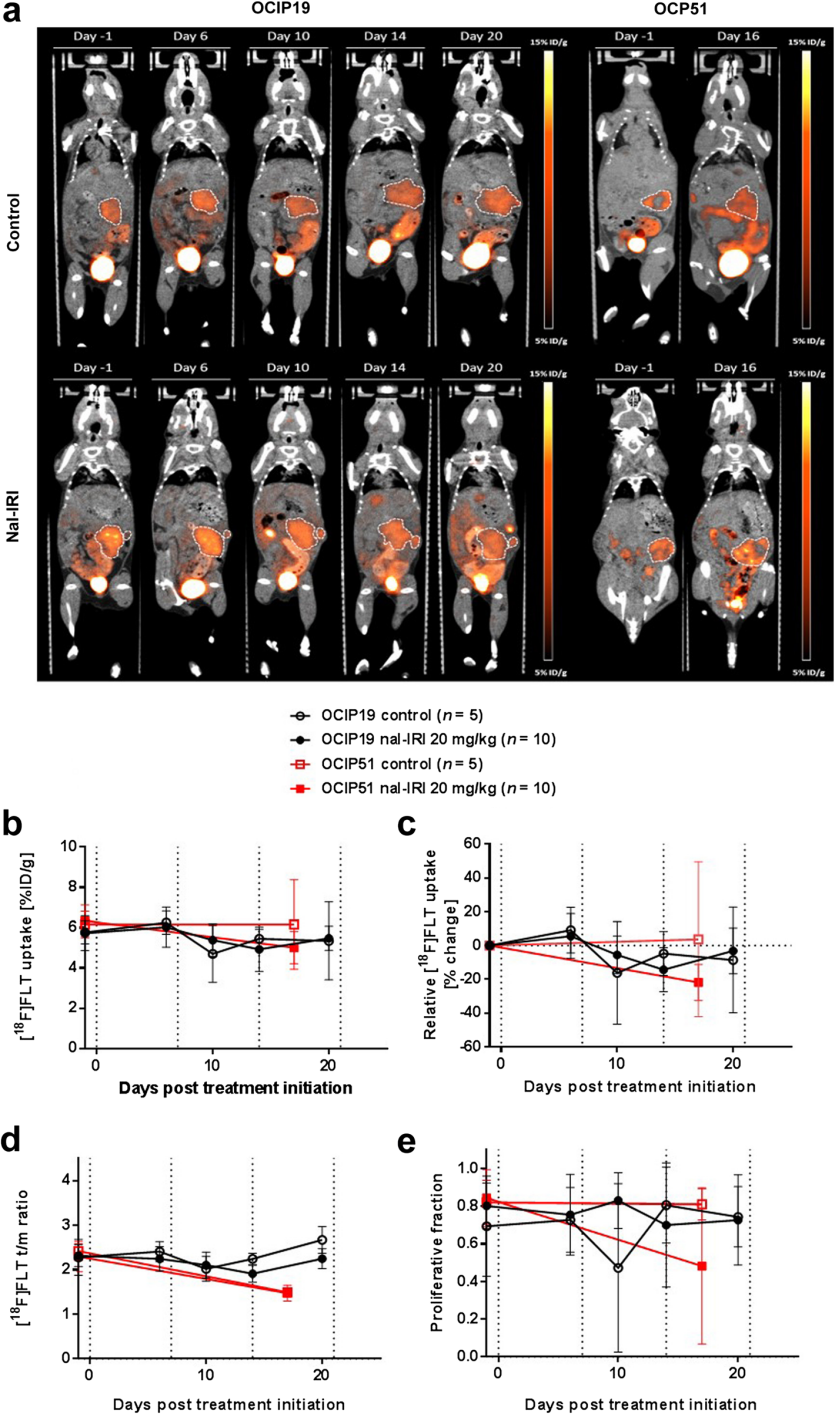
Longitudinal [^18^F]FLT-PET imaging. **a** Representative coronal 2D PET/CT images of a treated *versus* a control animal for each tumor model. Tumors are highlighted by the white dashed lines. **b** [^18^F]FLT uptake expressed as mean %ID/g calculated from the entire tumor volume and as **c** uptake percent change, compared with treatment initiation day 0. **d** [^18^F]FLT uptake expressed as tumor to muscle (t/m) ratio of the mean %ID/g. **e** Tumor proliferative fraction calculated from the PET data set. Dotted vertical lines indicate time points of nal-IRI treatment administration.

[^18^F]FLT-PET is used in the clinic as a prognostic imaging biomarker to evaluate patient response to radiotherapy and chemotherapy treatments [34–37]. For the OCIP51 model, [^18^F]FLT-PET was performed twice, once before the start of dosing and at day 16 post-treatment initiation, whereas for the OCIP19 model, [^18^F]FLT-PET was performed five times. Both tumor models showed [^18^F]FLT uptake at baseline (6.28 ± 0.70 %ID/g and 5.74 ± 0.74 %ID/g for OCIP51 and OCIP19, respectively). However, we did not observe any statistically significant difference in [^18^F]FLT uptake (*i.e., p* < 0.05) in terms of mean tumor uptake (%ID/g), percent change in mean tumor uptake, tumor/muscle ratio, or tumor proliferative fraction between the treated and control mice in both the OCIP19 and the OCIP51 groups (Fig. 4a–e). Despite the stark difference in disease progression rate between the two tumor models, no difference in [^18^F]FLT uptake was detected between the two models at any time point.

For the nal-IRI-treated OCIP51 animals, a reduction in [^18^F]FLT-PET tumor-to-muscle (t/m) signal ratio (t/m ratio, *p* < 0.0001) and proliferative fraction (*p* = 0.0273) was observed on day 16 post-treatment initiation compared with baseline (Fig. 4d, e). FLT and FLT_max_ measurements in the OCIP51 model also showed a significant decrease compared with baseline after treatment with nal-IRI that was not present in control tumors (Fig. 4b, Suppl. Fig. S5 in ESM). However, no statistically significant difference for the t/m ratio and proliferative fraction between treated and control animals was detected at the same time point (*p* > 0.5). Furthermore, t/m ratios were also reduced in control animals relative to baseline in this tumor model, although the early dropout of some of the control tumors (2/5) prior to the FLT measurement at day 16 may have biased the data.

### [^18^F]FLT-PET Imaging of Treatment-Induced Hematopoiesis

**Fig. 5:**
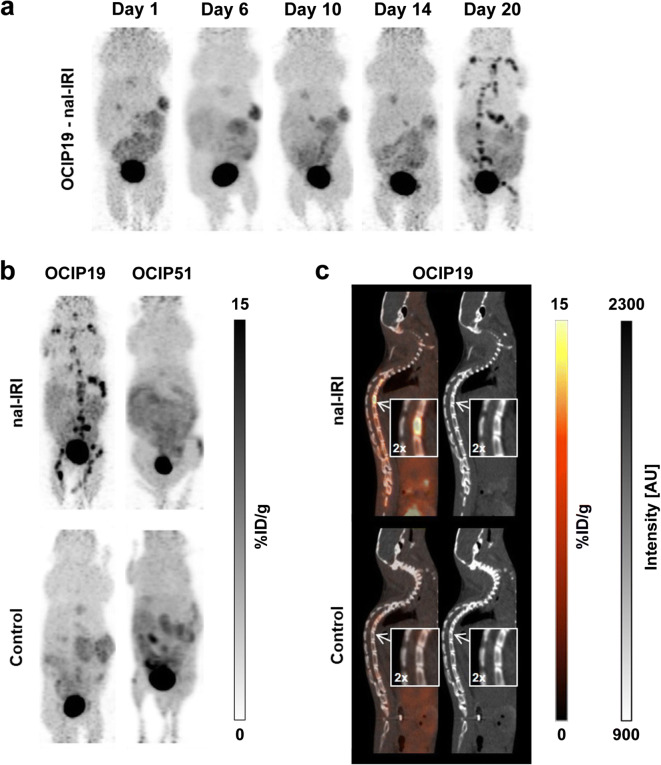
**a** Longitudinal coronal [^18^F]FLT-PET images from a representative mouse treated with nal-IRI. **b** Representative coronal [^18^F]FLT-PET images from treated and control OCIP19 and OCIP51 mice. [^18^F]FLT bone marrow and spleen uptake is only visible in the nal-IRI-treated OCIP19 mouse. **c** Representative [^18^F]FLT-PET-CT and CT images of a treated and a control OCIP19 mouse.

A surprising finding was that significant [^18^F]FLT uptake in the bone marrow and spleen was observed in nine out of the ten treated OCIP19 animals on day 20 post-treatment initiation (Fig. 5a), whereas no abnormal [^18^F]FLT uptake was observed in these same tissues in the untreated OCIP19 mice, nor in the treated or control OCIP51 mice, albeit only at 2 days post-dosing of cycle 3 (Fig. 5b). In the corresponding CT images, no sign of bone metastasis was observed (Fig. 5c). No similar signal was seen during the first dosing cycle or during the second dosing cycle (Fig. 5a).

### Tumor Weight and Morphology at the Study Endpoint

**Fig. 6:**
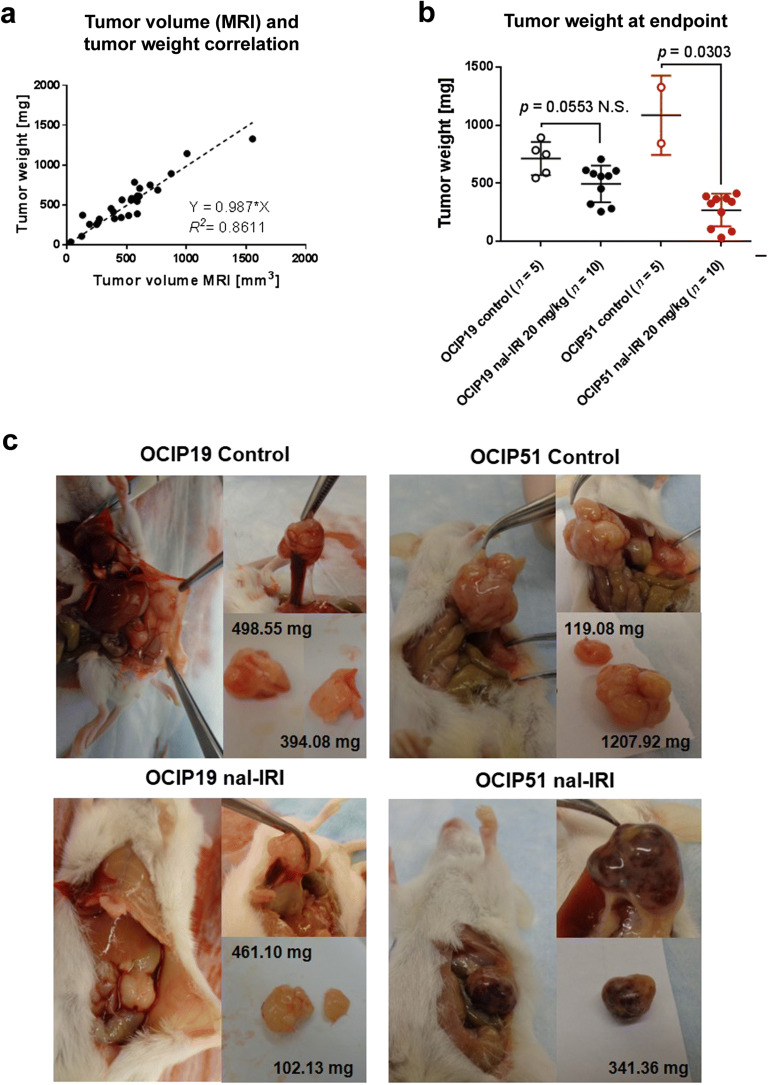
Tumor weight and morphology at the study endpoint. **a** Positive correlation between tumor weight (g) and tumor volume measured by MRI, at the study endpoint. **b** Tumor weight measured after resection showing significant difference between treated and control animals only in the OCIP51 group. **c** Representative images and corresponding tumor weights for the four groups, during tumor resection.

At the study endpoint, 22 days post-treatment initiation, and 24 h after the fourth and last dose of nal-IRI, OCIP19 and OCIP51 tumors were collected, visually inspected, and their weights recorded. The tumor weight showed a positive correlation with the tumor volume determined by MRI on the same day (*R*^2^ = 0.8611, *r* = 0.967, *p* < 0.0001) (Fig. 6a). Additionally, in agreement with the tumor volume changes following nal-IRI treatment reported in Fig. 2a, a statistically significant difference in the weight of the excised tumors was found between treated and control animals only in the OCIP51 model (Fig. 6b, *p* = 0.0303 for OCIP51 and *p* = 0.0553 for OCIP19). Furthermore, a larger difference in tumor weight was found between the treated and untreated tumors of the OCIP51 model compared with the OCIP19 model. Based on visual inspection (Fig. 6c), a major difference in morphology was seen in the OCIP51-treated tumors compared with their untreated counterparts. The treated OCIP51 tumors were highly vascularized with focal pockets filled with blood (Fig. 6c). Conversely, there was no gross morphology difference between the treated and untreated OCIP19 tumors.

## Discussion

In this study, we investigated the value of [^18^F]FAZA-PET in assessing the baseline levels of hypoxia in two orthotopic patient-derived tumor models of pancreatic cancer, OCIP19 and OCIP51, as well as in longitudinal monitoring of tumor hypoxia modulation following treatment with nal-IRI. [^18^F]FAZA-PET confirmed the classification of each model with OCIP19 as a low hypoxia tumor (baseline hypoxic fraction of 2 %) and the OCIP51 as a high hypoxia tumor (baseline hypoxic fraction of 71 %). [^18^F]FAZA-PET provided an early assessment of treatment activity for the OCP51 model in which a significant drop in tumor hypoxia was detected for the treated group 8 days before a significant decrease in tumor volume was measured. Similarly, our previously reported results for the HT29 colorectal carcinoma model, which had a relatively high baseline hypoxic fraction of approximately 45 %, showed that [^18^F]FAZA-PET detected a statistically significant difference in tumor hypoxia levels 9 days before a statistically significant change in tumor volume was measured between treated and control group [35]. Both studies support the role of [^18^F]FAZA-PET hypoxia quantification as an early treatment response imaging biomarker following nal-IRI chemotherapy and suggest that hypoxia modulation may be a mechanism of action worth further exploring.

Nal-IRI at 20 mg/kg provided some tumor control for the low hypoxia model, and, at the study end point, a significant difference in tumor volume was found between treated and control group. The treatment outcome was less obvious compared with that of the OCIP51 model, possibly due to the slow-growing and less-aggressive nature of the OCIP19 tumors. The treatment efficacy of nal-IRI did not demonstrate any detectable hypoxia-lowering effect in this tumor model. However, it is remarkable to see how much more effective nal-IRI is in treating the highly aggressive (and highly hypoxic) OCIP51 tumor model. In fact, OCIP51 tumors progressed to a similar tumor volume (500 mm^3^) ~ 40 days earlier compared with OCIP19. At the study endpoint, after four weekly doses of nal-IRI, the treated OCIP51 tumors were on average 28 % of the weight of the untreated controls, while the treated OCIP19 tumors were on average 67 % of the weight of their untreated counterparts. Moreover, a difference in morphology was seen between OCIP51 and OCIP19 tumors at the time of resection. Specifically, the treated OCIP51 tumors were highly vascularized and filled with pockets of blood, compared with the untreated OCIP51 group as well as the treated and untreated OCIP19 tumors. These morphological changes support our hypoxia measurements as vascularization is correlated to hypoxia reduction. These results might suggest that nal-IRI, perhaps similar to other therapeutics such as trastuzumab, might be able to improve its own delivery and possibly that of other therapeutics when given in combination [38, 39]. The synergistic effect of enhanced drug delivery and hypoxia reduction might play a key role in nal-IRI’s mechanism of action, particularly in highly hypoxic tumor models.

[^18^F]FLT-PET has been shown to be a good predictor of early response to both radiotherapy and chemotherapy as well as to concurrent chemo-radiotherapy [34, 40]. [^18^F]FLT is used as a surrogate for visualization and quantification of cellular proliferation, an intrinsic characteristic of malignant lesions [33, 41]. Clinical and preclinical studies evaluating the role of [^18^F]FLT in predicting treatment outcome have shown that in brain, lung, and breast cancers, there is a strong correlation between [^18^F]FLT uptake and the expression of the Ki-67 protein, a cellular marker for proliferation [42]. Only a limited number of studies have been published on the use of [^18^F]FLT in pancreatic cancer, and among those, only Challapalli et al. explored its use as a predictor of the therapeutic response, finding that the maximum standardized uptake value at 60 min post-injection (SUV_60_,_max_) was predictive of disease progression but not of survival [41]. In our study, we observed no change in overall [^18^F]FLT uptake between control and nal-IRI-treated tumors over time in either the OCIP51 or OCIP19 tumors, despite the effectiveness of the treatment. However, treatment effects in the OCIP51 tumor model were confirmed by paired comparison of FLT, FLT t/m, and FLT_max_ levels in lesions compared with their respective baseline. The unchanged [^18^F]FLT uptake could be caused by a number of factors. For example, previous studies reported that [^18^F]FLT uptake is dependent on tumor thymidine levels, vascularity, and use of *de novo* or salvage DNA synthesis pathways [43]. Various pancreatic cancer models have been reported to express high levels of thymidylate synthase responsible for the *de novo* biosynthesis of dTMP [44]. In addition, response assessment has to be performed at an adequate time interval [31, 43]. The lack of a significant difference observed in the [^18^F]FLT uptake between treated and control animals in either tumor model thus cautions the use of [^18^F]FLT-PET as a surrogate imaging marker for cellular proliferation and ultimately for treatment response assessment.

Our [^18^F]FLT-PET results also showed bone marrow and spleen [^18^F]FLT uptake in nine out of ten treated animals after three weekly doses of nal-IRI, but not in control animals thus excluding a batch effect of the tracer. CT images did not show signs of bone metastasis, suggesting that, instead, the [^18^F]FLT-PET signal might originate from the proliferating cells in the bone marrow and spleen. This observation will be further explored and characterized.

## Conclusions

Results from this study support the usefulness of [^18^F]FAZA-PET for evaluation of tumor hypoxia during anti-cancer therapy with nal-IRI, as a means to provide early assessment of treatment activity. These results also support the use and continued exploration of nal-IRI in pancreatic cancer.

## Electronic Supplementary Material

ESM 1 (PDF 550 kb)
