# Supplementary material for: Longitudinal PET Imaging to Monitor Treatment Efficacy by Liposomal Irinotecan in Orthotopic Patient-Derived Pancreatic Tumor Models of High and Low Hypoxia
Source: Mol Imaging Biol. 2019 Sep 3;22(3):653–64. doi: 10.1007/s11307-019-01374-x (PMC7782415; doi:10.1007/s11307-019-01374-x)
Supplement: Supplementary file 1 — (PDF 550 kb) [file 11307_2019_1374_MOESM1_ESM.pdf]

Electronic Supplementary Material

Longitudinal PET Imaging to Monitor Treatment Efficacy by Liposomal Irinotecan in Orthotopic Patient-Derived Pancreatic Tumor Models of High and Low Hypoxia

Journal: Molecular Imaging and Biology

Manuela Ventura[1], Nicholas Bernards[1], Raquel De Souza[1†], Inga B. Fricke[1], Bart S. Hendriks[2†], Jonathan B. Fitzgerald[2†], Helen Lee[2†], Stephan G. Klinz[2†, 3] and Jinzi Zheng[1,4]

[1] TECHNA Institute for the Advancement of Technology for Health, University Health Network, Toronto, Ontario, Canada.

[2] Merrimack Pharmaceuticals, Inc., Cambridge, MA, USA

[3] Ipsen Bioscience, Cambridge, MA, USA

[4] Institute of Biomaterials and Biomedical Engineering, University of Toronto, Ontario, Canada.

†Affiliation at the time of study.

Running Title:

Nal-IRI efficacy in high and low hypoxic pancreatic tumors

Corresponding Author:

Jinzi Zheng, PhD

101 College Street, Room 7-302

Toronto, Ontario, Canada M5G 1L7

Tel: +1 (416) 581 7790

Fax: +1 (416) 506 1828

Email: jinzi.zheng@rmp.uhn.on.ca

Supplementary Materials and Methods

Animal Models

All animal studies were approved by the University Health Network (UHN) Animal Care Committee and adhere to the ethical guidelines of the Canadian Council on Animal Care. The animals were acclimatized for 1 week before use and monitored daily throughout the study. The mice were housed in groups of 5 per cage in a limited access area, at a mean temperature of 19-21°C and a humidity of 40%–60%. Food and water were provided *ad libitum*. Fragments of the patient-derived pancreatic tumors OCIP51 and OCIP19 (UHN Tumor Tissue Bank, Toronto) were implanted orthotopically [Ref 12 in the main text] into 6 to 8-week-old female NOD/SCID mice (Ontario Cancer Institute, Toronto, Canada). Based on the MR images, tumors that had invaded the subcutaneous space, which size was significantly different from the average and which presented morphological anomalies at a visual inspection were excluded from the study.

In Vivo Imaging

MR imaging was performed on a 1T-MRI (M3, Aspect Imaging, Shoham, Israel) with the following parameters: T2-weighted Fast Spin Echo: scan time = 8:24 min, echo time/repetition time = 57.3 ms / 4500 ms, number of excitations = 6, voxel size = 156 µm x 156 µm x 1mm slice thickness, up to 22 slices to ensure full coverage of the tumors. [^{18}F]FAZA and [^{18}F]FLT were produced by CanProbe (Hamilton, ON, Canada) with an average radiochemical purity of 97.9 ± 2.1% for [^{18}F]FAZA (n = 9), and 100.0 ± 0.0% for [^{18}F]FLT (n = 6). PET imaging was performed on a Focus 220 preclinical PET scanner (Siemens, Nashville, TN). Each [^{18}F]FAZA-PET acquisition was performed at 2 h post tracer administration (average injected activity 9.04 ± 0.91 MBq, 0.452 ± 0.46 Mbq/g, calculated over nine imaging sessions, and 13 to 15 mice per session) and consisted of a 20 min emission scan followed by an 8 min Co-57 transmission scan for

attenuation and scatter correction. Each [^{18}F]FLT-PET acquisition was performed at 1 h post-tracer administration (average injected activity 8.64 ± 1.15 MBq, 0.420 ± 0.65 MBq/g calculated over seven imaging sessions, and 13 to 15 mice per session) and consisted of a 10 min emission scan followed by an 8 min ^{57}Co transmission scan. Immediately after PET imaging, a microCT scan (GE Healthcare Locus Ultra, Little Chalfont, UK; 80 kVp, 50 mA, radiation dose per scan 90 mGy) was performed for anatomical reference. PET images were reconstructed using a maximum a posteriori (MAP) algorithm with voxel size of 0.146 x 0.146 x 0.796 mm^3. Please refer to Figure S1 (ESM) for a schematics of the *in vivo* imaging timeline.

Supplementary Table and Figures

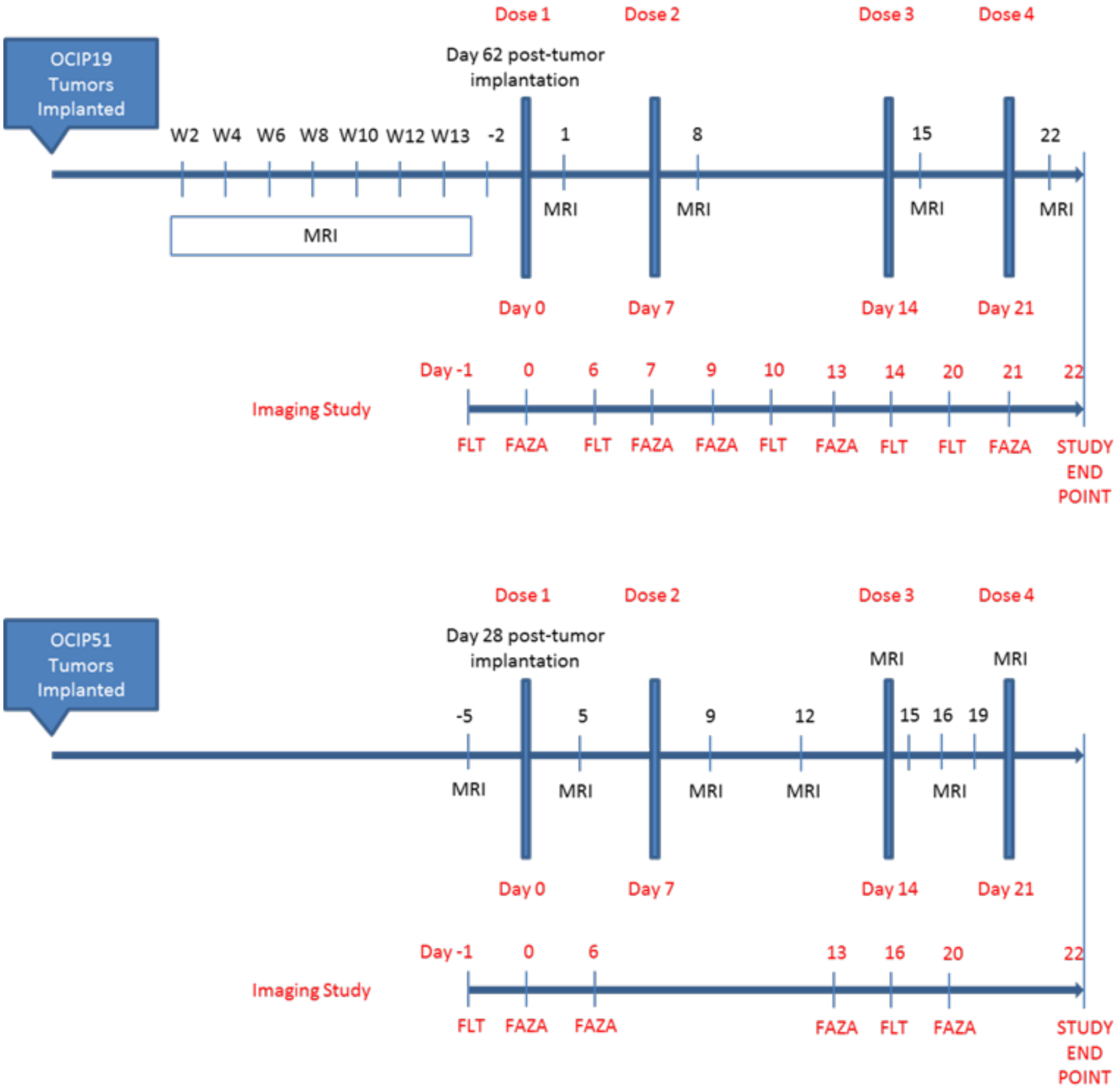

Figure S1. Schematics of the *in vivo* imaging timeline and therapeutic scheme.

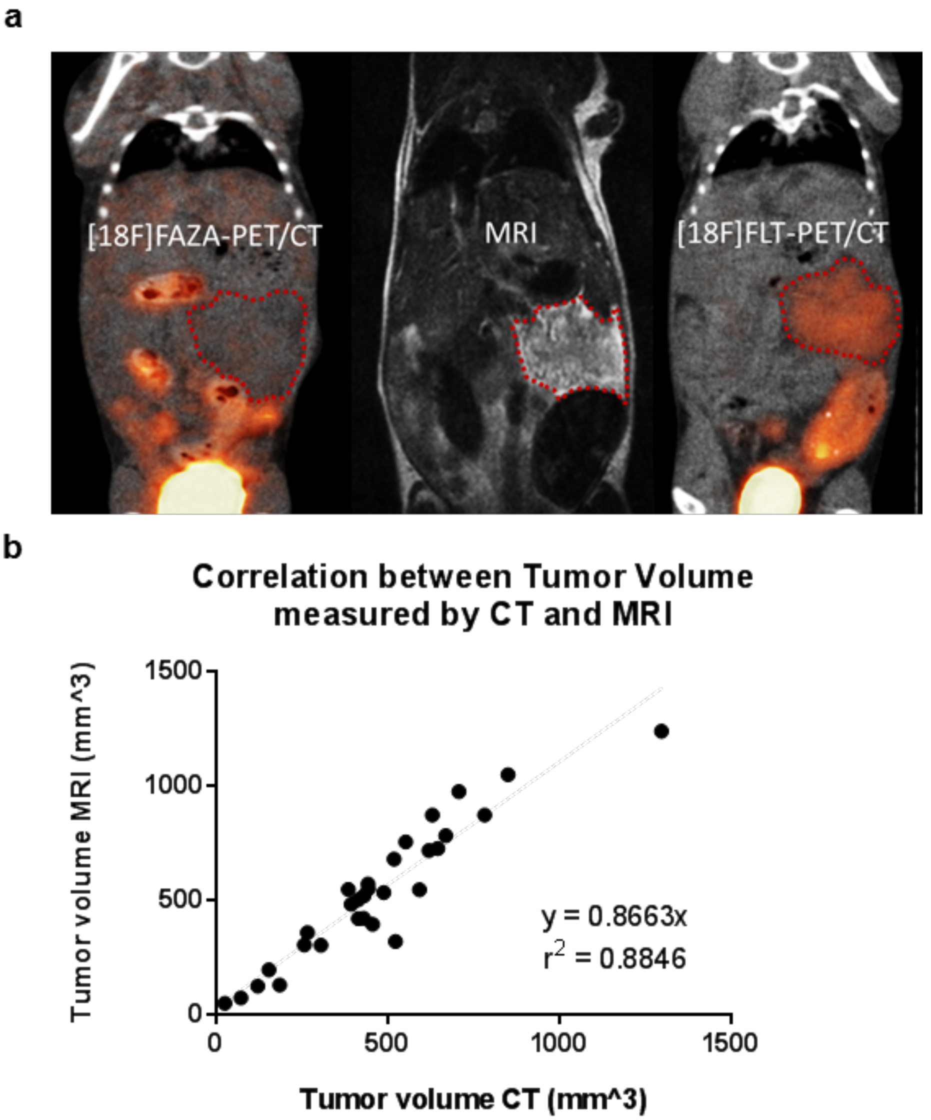

Figure S2. CT- and MRI-based tumor volume measurements. (a) Representative [18F]FAZA-PET/CT, MRI, and [18F]FLT-PET/CT 2D images of a OCIP19 tumor-bearing mouse. The ROI, used for tumor volume quantification, is shown as a red, dotted line. (b) Correlation between tumor volumes measured by MRI and PET/CT (p < 0.0001).

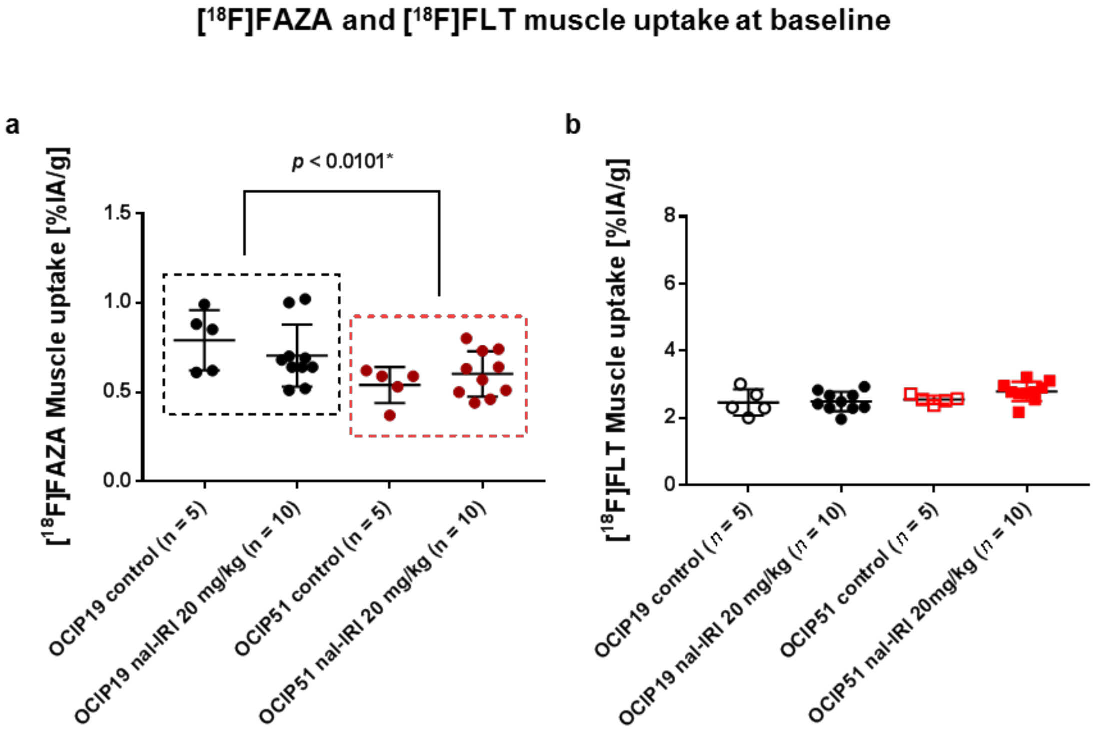

Figure S3. (a) [^{18}F]FAZA and (b) [^{18}F]FLT muscle uptake at the study baseline.

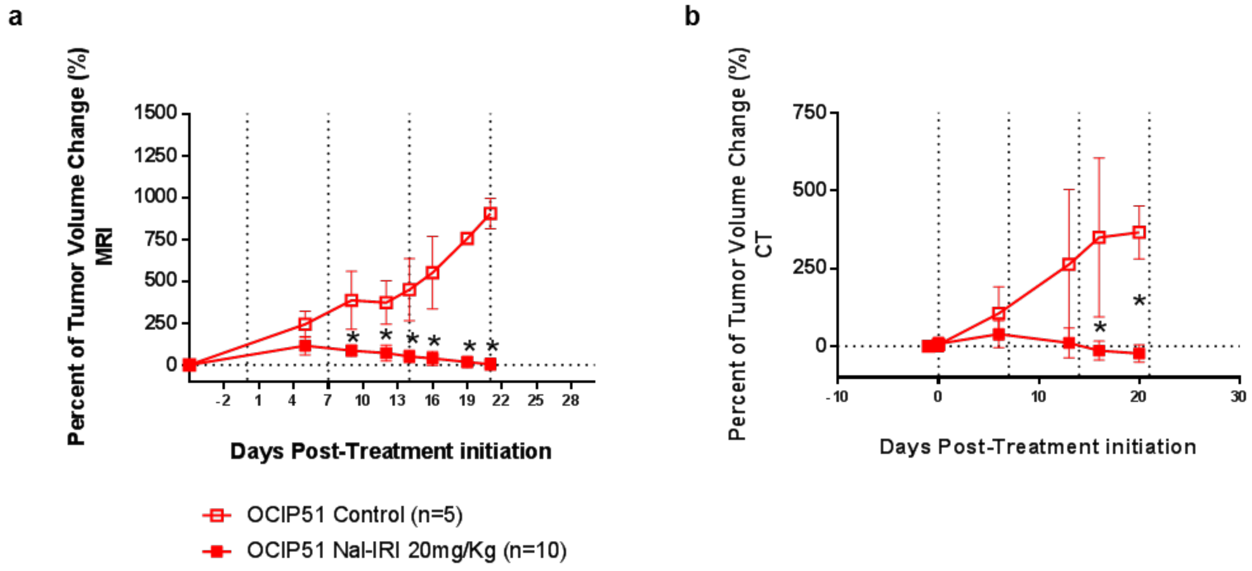

Figure S4. Relative changes in tumor volumes obtained with MRI and PET/CT imaging modalities. Relative changes were calculated from volume measurements obtained with (a) MRI or (b) PET/CT. Asterisks denote a statistically significant difference between the nal-IRI treated group and the control group. Dotted vertical lines indicate time points of nal-IRI treatment administration.

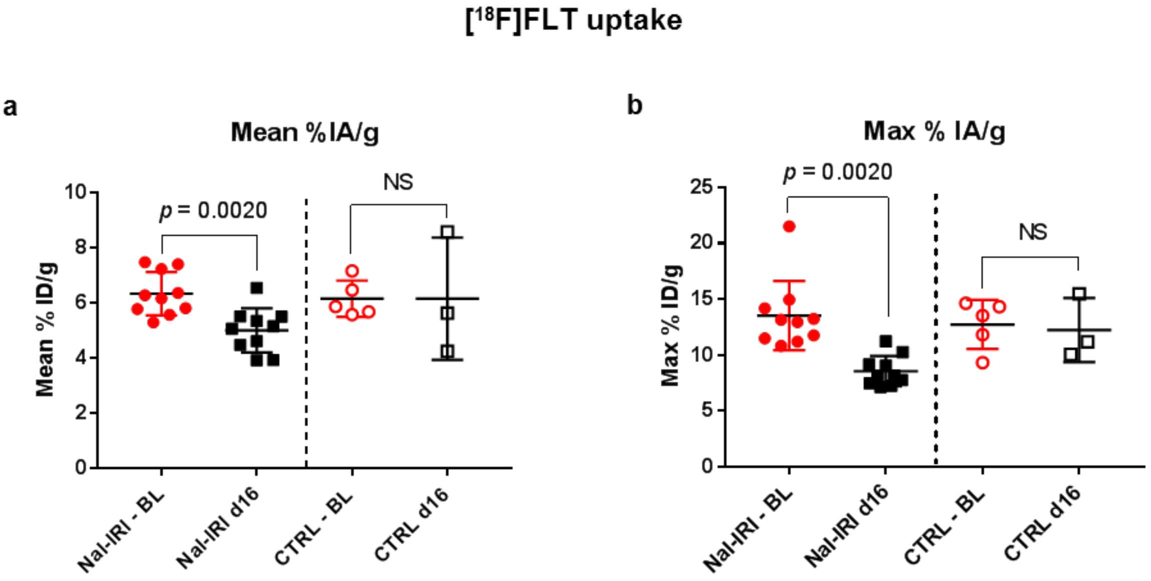

Figure S5. Comparison between [^{18}F]FLT uptake in OCIP51 tumors, pre-treatment initiation, at the study baseline (BL), and 16 days post-treatment. [^{18}F]FLT uptake is expressed as mean % IA/g calculated from the entire tumor volume (A) or as (B) max % IA/g . The statistical differences between time points was calculated with a paired, non-parametric, Wilcoxon test.
